# Impact of low-grade inflammation on neurological outcomes in patients with acute pontine infarction: a retrospective cohort study

**DOI:** 10.3389/fimmu.2025.1744943

**Published:** 2026-01-13

**Authors:** Junyu Zhu, Hai Zhou, Jierong Mo, Tianen Zhou, Jun Jiang, Zhiwen Zhang

**Affiliations:** 1 Department of Emergency, The First People’s Hospital of Foshan (Foshan Hospital Affiliated to Southern University of Science and Technology), School of Medicine, Southern University of Science and Technology, Guangdong, China; 2 Central Laboratory, Yunfu People’s Hospital, Yunfu, Guangdong, China; 3 Department of Medical Affairs, The First People’s Hospital of Foshan, (Foshan Hospital Affiliated to Southern University of Science and Technology), School of Medicine, Southern University of Science and Technology, Guangdong, China

**Keywords:** acute pontine infarction, inflammatory markers, low-grade inflammation, neurological outcomes, prognosis

## Abstract

**Background:**

Acute pontine infarction significantly impacts neurological function and prognosis. Recent studies suggest that low-grade inflammation (LGI) may play a critical role in influencing recovery outcomes. However, the relationship between LGI and neurological prognosis in acute pontine infarction remains underexplored.

**Objectives:**

This study aims to investigate the correlation between LGI and neurological outcomes in patients with acute pontine infarction over 90 days post-stroke.

**Methods:**

A retrospective cohort study was conducted involving 502 patients diagnosed with acute pontine infarction at The First People’s Hospital of Foshan between January 1, 2015, and July 14, 2022. Clinical data, inflammatory markers (CRP, WBC, NLR, PLT), and neurological function assessed via NIHSS scores were collected and analyzed.

**Results:**

Elevated levels of inflammatory markers were significantly associated with poorer neurological outcomes. The LGI scores correlated positively with NIHSS scores at discharge and 90 days post-stroke, indicating that higher LGI is predictive of impaired recovery.

**Conclusion:**

This study demonstrates that low-grade inflammation is an important predictor of neurological outcomes in acute pontine infarction patients. Incorporating LGI assessments into clinical practice may enhance prognostic accuracy and inform treatment strategies.

## Introduction

Acute pontine infarction, as a severe type of cerebrovascular event, often leads to significant functional impairment and high mortality rates (1). The definition and management of this condition not only affect the recovery process of patients but also directly relate to their long-term prognosis (2). In recent years, an increasing body of research has suggested that low-grade inflammation (LGI) plays an important role in the neurological prognosis of patients after acute stroke. However, there remains a considerable gap in understanding the specific relationship between LGI and acute pontine infarction (3). This study aims to investigate the impact of LGI on neurological outcomes within 90 days following acute pontine infarction, providing data support for clinical decision-making.

The pathological changes associated with cerebral infarction are typically accompanied by an inflammatory response in the body. Studies indicate that low-grade inflammation is closely linked to the onset of many cardiovascular and cerebrovascular diseases (4, 5). Research has shown that after an acute ischemic stroke, the inflammatory response not only potentially exacerbates neurological injury during the initial phase but may also affect overall prognosis by influencing endothelial function, microvascular permeability, and thrombosis formation (6, 7). Therefore, exploring the role of LGI in patients with acute pontine infarction is vital for improving patient management and developing personalized treatment plans.

Existing studies predominantly focus on mechanisms of acute ischemic stroke and their associations with short-term outcomes, while the influence of LGI, particularly in the context of pontine infarction, is poorly understood. The literature indicates that elevated inflammatory markers such as C-reactive protein (CRP) correlate with poor prognoses in acute stroke patients, suggesting the potential of LGI as a viable biomarker for clinical applications (8, 9). Thus, conducting research on LGI is particularly important, especially in assessing recurrence risk, enhancing clinical intervention strategies, and reducing recurrence rates. Accordingly, this study will investigate the impact of LGI on the 90-day prognosis of patients with acute pontine infarction, advancing the research on acute stroke treatment (10, 11).

The primary goal of this study is to systematically assess the neurological recovery status of 502 patients diagnosed with acute pontine infarction within 90 days using a retrospective cohort design. We will employ various inflammatory markers, including CRP, white blood cell count (WBC), neutrophil-to-lymphocyte ratio (NLR), and platelet count (PLT), to develop a low-grade inflammation (LGI) scoring system and conduct comprehensive analyses based on clinical data. By correlating LGI with clinical characteristics and prognostic outcomes, this study aims to explore LGI’s predictive value for neurological recovery in acute pontine infarction patients.

Additionally, we will use advanced regression analyses and machine learning models to quantify the key factors influencing prognosis and establish the relationship between LGI and various risk factors. This approach aims to provide more precise predictive tools for clinical practice. As personalized medicine continues to advance, understanding the connection between LGI and prognosis will lead to improved management strategies, enhancing patient quality of life and reducing the risk of subsequent cerebrovascular events. Ultimately, our investigation into LGI’s impact will offer valuable insights for the field of neurology.

## Methods

### Study population

This retrospective study enrolled 502 patients diagnosed with acute pontine infarction at The First People’s Hospital of Foshan between January 1, 2015, and July 14, 2022. The inclusion criteria were strictly adhered to ensure the specificity and relevance of the chosen patient cohort. Eligible patients were those who met the criteria outlined in the “2018 Guidelines for the Diagnosis and Treatment of Acute Ischemic Stroke in China.” Participants had to be first-time cases of pontine infarction or have a previous history of stroke without persistent neurological deficits. All subjects were required to be at least 18 years old to ensure they could provide informed consent. MRI scans were mandatory for all patients to confirm lesions in the pontine region, thereby ensuring diagnostic accuracy.

**Figure 1:**
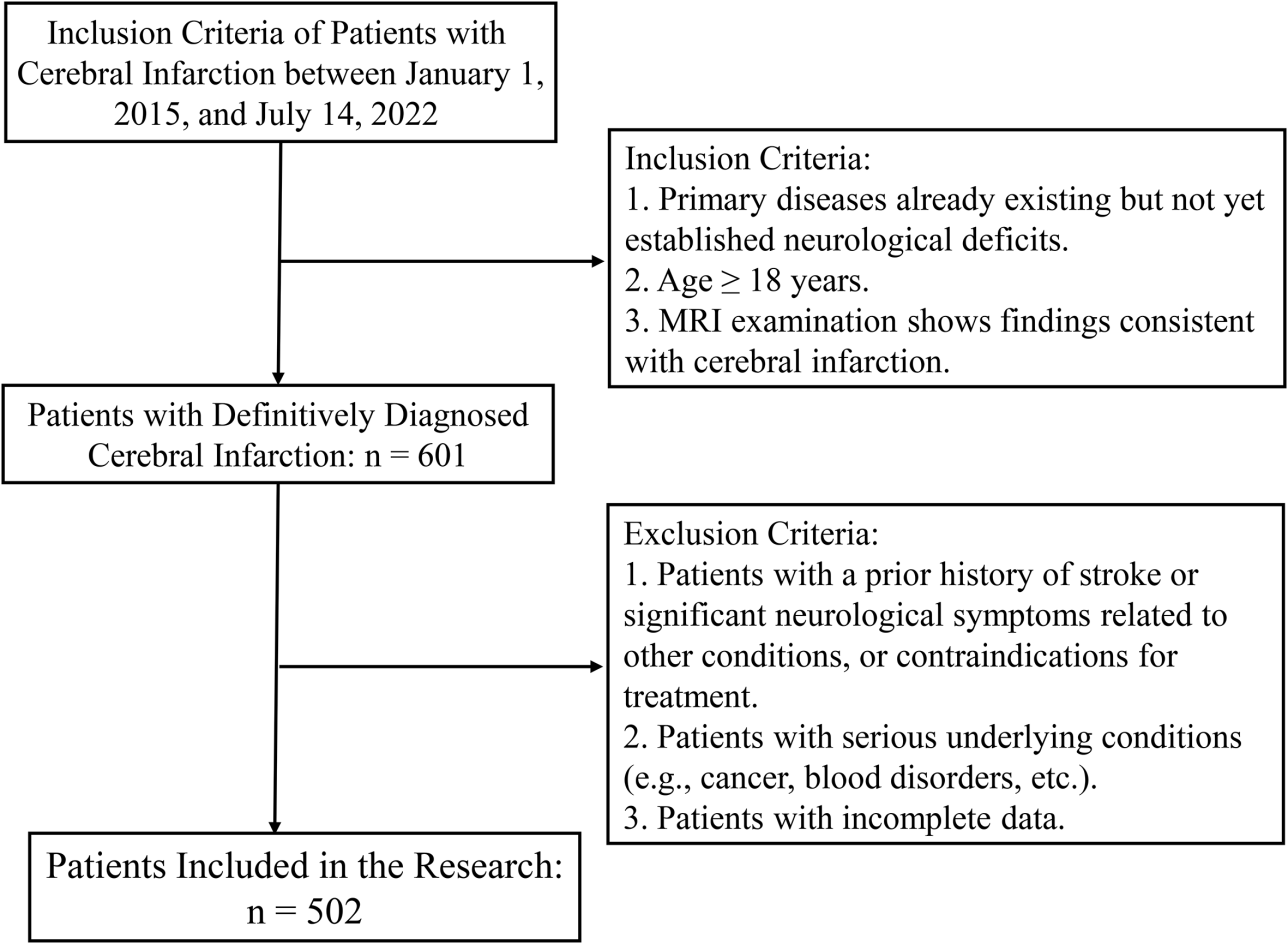
Patient recruitment and exclusion flowchart for acute pontine infarction study.

Exclusion criteria were meticulously designed to eliminate potential confounding factors. Patients presenting with acute infectious symptoms or those who had received anti-infective medications, corticosteroids, or immunosuppressants within one week prior to symptom onset were excluded. Furthermore, individuals with significant comorbidities, such as malignant tumors, hematological diseases, chronic inflammatory conditions, autoimmune diseases, or severe hepatic and renal insufficiency, were also omitted to maintain the reliability of the outcomes. Finally, patients lacking sufficient clinical data or unable to adhere to follow-up assessments were excluded from the study (**Figure 1**).

### Inclusion and exclusion criteria

#### Inclusion criteria

Patients were eligible for inclusion in this study if they met the following criteria:

1. Confirmed Diagnosis: Patients with acute pontine infarction confirmed by brain imaging (CT or MRI) within 72 hours of symptom onset.
2. Clinical Presentation: Patients presenting with neurological deficits consistent with acute pontine infarction, such as cranial nerve dysfunction, limb weakness, or altered level of consciousness.
3. Age: Patients aged 18 years and older.
4. Informed Consent: Patients or their legal representatives provided informed consent to participate in the study.

#### Exclusion criteria

Patients were excluded from the study if they met any of the following criteria:

1. Pre-existing Neurological Disorders: Patients with a history of significant neurological disorders (e.g., prior stroke, major head trauma) that affect the interpretation of neurological deficits.
2. Non-vascular Causes: Patients whose symptoms were attributed to causes other than acute ischemic stroke, such as hemorrhagic stroke or tumors.
3. Severe Comorbidities: Patients with severe comorbid medical conditions that could interfere with treatment or prognosis (e.g., terminal cancer, severe hepatic or renal failure).
4. Withdrawal of Consent: Patients who withdrew consent at any time during the study.

This study included all eligible cases of acute pontine infarction that met the above criteria in the analysis. We ensured a comprehensive review of patient records to confirm the diagnosis and evaluate the appropriateness of inclusion and exclusion based on the defined criteria.

### Research methods

#### Clinical data collection and testing

Comprehensive information on both internal and external clinical factors was collected from all patients, including basic personal information (e.g., name, sex, age). Patient histories were recorded in detail, with a focus on lifestyle factors such as smoking and alcohol consumption, alongside the presence of comorbidities including hypertension, diabetes, coronary heart disease, and atrial fibrillation. Hypertension management was assessed by evaluating patient adherence to medication and whether blood pressure was well-controlled, with control defined as systolic blood pressure ≤140 mmHg and/or diastolic blood pressure ≤90 mmHg. Diabetes management was evaluated through fasting blood glucose levels and adherence to diabetes medications.

To obtain data on the inflammatory status of patients, blood samples were collected immediately before the commencement of treatment. These samples were sent to the laboratory and analyzed using an automatic cell analyzer (SYSMEX-XN9000) to measure various hematological parameters, including:

WBC, Neutrophil and Lymphocyte Counts, NLR, PLT, CRP, FIB, Coagulation Times, including PT, APTT, and TT, Fasting Blood Glucose, HbA1c, Lipid Profiles, including TC, TG, LDL-C, and HDL-C.

These tests provided a comprehensive assessment of each patient’s health status and the inflammatory response associated with acute pontine infarction.

### Scoring system

In constructing the LGI scoring system, patients were classified based on specific inflammatory markers. This scoring system utilized the quartiles of laboratory data collected, particularly focusing on CRP, WBC, NLR, and PLT. Quartile thresholds established for each biomarker were used to define low, medium, and high levels. For example, CRP levels were categorized into four groups: Q1 (lowest), Q2, Q3, and Q4 (highest). Each quartile was assigned a score from 1 to 4, with higher scores representing greater levels of inflammation. The total LGI score was calculated by summing the quartile scores of the four biomarkers, resulting in a range from 4 to 16, thereby reflecting varying degrees of low-grade inflammation.

Subsequent evaluations were conducted through systematic assessments via telephone interviews or outpatient follow-ups. During these follow-ups, the incidence of disease recurrence was assessed, and patients’ scores on the National Institutes of Health Stroke Scale (NIHSS) were measured at discharge and again 90 days post-discharge. An NIHSS score greater than 5 was considered indicative of poor prognosis, providing a quantifiable metric for evaluating patients’ functional status following a stroke.

### Statistical analysis

In this study, all statistical analyses were conducted using R (version 4.3.1). Descriptive statistics were utilized to summarize the baseline characteristics of the patients, including means ± SD for continuous variables and frequencies and percentages for categorical variables. Independent samples t-tests were employed to compare continuous variables between different groups, while chi-square tests were used for categorical variable comparisons.

Further statistical analyses included:

1. Linear Regression Analysis: This was performed to assess the relationship between LGI and the NIHSS score at discharge. The model controlled for confounding variables such as age, gender, diabetes management, and hypertension control.
2. Logistic Regression Analysis: Used to examine the association of LGI with recurrence status and prognostic outcomes (90-day NIHSS score). This model considered other factors that might affect recurrence rates, such as a history of prior stroke and gender.
3. Multiple Regression Analysis: Explored the interaction effects between LGI and clinical variables on the NIHSS scores. This enabled us to identify differences in the impact of low-grade inflammation between male and female patients.
4. Machine Learning Models: Including logistic regression, random forests, and XGBoost, were employed to evaluate the predictive ability of primary clinical characteristics on patient outcomes. Model performance was assessed through calculations of AUC and F1 scores.

The statistical analyses conducted began with descriptive statistics to elucidate baseline characteristics across groups, employing t-tests or chi-square tests as appropriate to assess differences in proportions and means. Clinical variables showed an assortment of significant differences between the two prognostic groups, notably in hypertension prevalence, diabetes management, atrial fibrillation, previous history of stroke, and coronary artery disease. These analyses revealed that patients in the poor prognosis cohort had significantly elevated inflammatory markers, including CRP, while other markers like NLR trended in a similar direction, emphasizing a notable role for inflammation in the acute neurological injury context.

Correlation analysis aims to assess the strength and direction of relationships between variables, commonly using Pearson correlation coefficients or Spearman rank correlation coefficients. The Pearson correlation coefficient is suited for normally distributed data and reflects linear relationships, with values ranging from -1 to 1, where 1 indicates a perfect positive correlation, -1 indicates a perfect negative correlation, and 0 indicates no correlation. The Spearman rank correlation coefficient is suitable for non-normally distributed data or ordinal variables, measuring associations based on ranks rather than raw data.

In this study, clinical parameter data related to NIHSS scores was collected, including patient demographics, medical history, treatment responses, and relevant biochemical markers. The correlation coefficient was calculated for each pair of variables to evaluate their relationships. To assess the statistical significance of the correlations, hypothesis testing was conducted, with a significance level set (e.g., α = 0.05). If the p-value of the correlation coefficient is below this significance level, it indicates that a significant correlation exists between the two variables. Potential confounding variables were considered to ensure the accuracy and credibility of the results. Through these analyses, researchers gain valuable insights into the relationships among various clinical factors and their impact on patient outcomes.

A robust linear regression analysis was employed to identify predictors related to the discharge NIHSS score among subjects with acute pontine infarction. This analytic framework incorporated various clinical parameters, including low-grade inflammation (LGI) levels, age, sex, medical histories, and laboratory values, enriching the model’s predictive capacity. The regression model allowed for estimating coefficients that indicated the strength of associations with the discharge NIHSS score, despite some variables not reaching statistical significance. To ensure the reliability of the results, we assessed potential multicollinearity among predictors using Variance Inflation Factors (VIF) and applied robust regression techniques, such as Huber weighting, to mitigate the influence of outliers. The model’s performance was evaluated through residual analysis and R-squared values to ascertain the variance explained by the predictors. Statistical significance was determined with a threshold set at p < 0.05. The analysis provided valuable insights into the impact of LGI on neurological recovery, revealing that each unit increase in LGI corresponds to a notable rise in NIHSS scores, suggesting impaired neurological recovery and highlighting the importance of addressing inflammation in clinical practice.

Furthermore, a logistic regression model assessed the impact of LGI on recurrence status and overall prognostic outcome, revealing that while LGI alone did not significantly impact recurrence, it held a positive correlation with overall prognosis. Other factors such as male sex and history of diabetes mellitus emerged as significant predictors for recurrence, thereby informing clinical considerations in managing patients with pontine infarction.

For a nuanced exploration of relationships between LGI and clinical variables, interaction terms were examined within multiple regression frameworks. The significant interaction between LGI and sex indicated that inflammatory effects were notably pronounced in male patients, highlighting the necessity of tailoring treatment approaches based on gender-specific physiology. Additionally, factors such as BMI, smoking status, and alcohol consumption were integrated to analyze their mediating roles in the relationship between LGI and stroke severity, thus ensuring a comprehensive model of patient health outcomes.

Mediation analysis was conducted to explore how health-related factors LGI and consequent patient outcomes. This procedure clarified the indirect effects of lifestyle factors like smoking and alcohol consumption on inflammation levels and overall prognosis, revealing critical insights for preventive strategies targeting modifiable risk factors in stroke patients. By quantitatively assessing these relationships, we were able to delineate the pathways through which lifestyle factors contribute to inflammation and patient outcomes, thereby highlighting areas where intervention could significantly benefit health outcomes.

To complement our assessment, machine learning models were utilized to evaluate and predict patient outcomes based on these variables, showcasing the potential of employing advanced analytic techniques in clinical settings. We systematically evaluated several models, including Logistic Regression, Random Forest, XGBoost, and Ensemble models, for their predictive powers, employing metrics such as the area under the curve (AUC) and F1 scores to measure accuracy and model performance. The ensemble model produced the highest predictive accuracy, reinforcing the importance of critical features, such as LGI, diabetes history, and other clinical variables, as central determinants of patient outcomes.

A crucial aspect of our methodology was the application of causal inference techniques to delineate direct and indirect effects among the variables studied. The use of a DAG enabled us to visualize and understand the temporal sequences and causal relationships between health-related factors, LGI, and patient outcomes. This graphical representation helped us explicitly control for confounding factors, ensuring that our analysis focused on the actual mechanisms through which these lifestyle factors affect inflammation levels and health outcomes rather than mere associations.

Furthermore, by integrating mediation analysis with machine learning approaches, we were able to not only observe the direct and indirect pathways of influence but also validate these pathways using predictive modeling. The insights gained from this combination of methods underscore the importance of considering both the statistical relationships and the underlying causal mechanisms when evaluating the impact of modifiable risk factors on health outcomes. This multifaceted approach provides a more robust framework for developing strategies aimed at improving patient care and outcomes in clinical practice, particularly for stroke patients.

### Ethical considerations

This study adhered to the ethical principles outlined in the Declaration of Helsinki and followed the protocol approved by the Institutional Review Board (IRB) of The First People’s Hospital of Foshan (Ethical Review Approval Number: 2024-105). All patients provided written informed consent before data collection. Throughout the research process, patient privacy and confidentiality were maintained, with all identifying information anonymized in the dataset to ensure compliance with research ethics standards. All results were reported in aggregate form to protect patient confidentiality.

By employing rigorous statistical analyses and adhering to ethical guidelines, this methodology aims to elucidate the association between low-grade inflammation and neurological outcomes in patients with acute pontine infarction, thereby contributing to a broader understanding of the inflammatory processes involved in cerebrovascular events.

## Results

### Low-grade inflammation scores in predicting neurological outcomes in patients with pontine infarction

A total of 502 patients with pontine infarction were evaluated based on their outcomes at 90 days, classified into good prognosis (non-poor prognosis, n=454) and poor prognosis (n=48) (**Table 1**). There were no significant differences in sex distribution, with males constituting 66.3% of the total cohort. Age means were comparable, with an overall mean of 64.6 ± 11.4 years, and although the poor prognosis group was older (66.4 ± 10.3 years), this was not statistically significant (p=0.242). Smoking status and alcohol consumption did not differ significantly between groups.

**Table 1:** Comparison of demographics and clinical variables between patients with good and poor prognosis at 90 days post-stroke.

Variable	Total (n=502)	Control(N = 454)	Poor prognosis (n=48)	P-value
Sex, *n* (%)				0.71
Female	169 (33.665)	154 (33.921)	15 (31.250)	
Male	333 (66.335)	300 (66.079)	33 (68.750)	
Age, Mean ± SD	64.604 ± 11.398	64.410 ± 11.500	66.438 ± 10.310	0.24
Smoking Status, *n* (%)				0.85
None	360 (71.713)	325 (71.586)	35 (72.917)	
Yes	142 (28.287)	129 (28.414)	13 (27.083)	
Alcohol Consumption, *n* (%)				0.37
None	440 (87.649)	396 (87.225)	44 (91.667)	
Yes	62 (12.351)	58 (12.775)	4 (8.333)	
Hypertension, *n* (%)				0.07
None	276 (54.980)	245 (53.965)	31 (64.583)	
Good Control	124 (24.701)	111 (24.449)	13 (27.083)	
Poor Control	102 (20.319)	98 (21.586)	4 (8.333)	
Diabetes Mellitus, *n* (%)				<0.01
None	418 (83.267)	385 (84.802)	33 (68.750)	
Good Control	51 (10.159)	43 (9.471)	8 (16.667)	
Poor Control	33 (6.574)	26 (5.727)	7 (14.583)	
Atrial Fibrillation, *n* (%)				0.05
None	493 (98.207)	448 (98.678)	45 (93.750)	
Yes	9 (1.793)	6 (1.322)	3 (6.250)	
Antiplatelet History, *n* (%)				0.03
None	494 (98.406)	449 (98.899)	45 (93.750)	
Yes	8 (1.594)	5 (1.101)	3 (6.250)	
History of Stroke, *n* (%)				0.03
None	429 (85.458)	393 (86.564)	36 (75.000)	
Yes	73 (14.542)	61 (13.436)	12 (25.000)	
History of Cancer, *n* (%)				1
None	500 (99.602)	452 (99.559)	48 (100.000)	
Yes	2 (0.398)	2 (0.441)	0 (0.000)	
History of Gout, *n* (%)				0.61
None	492 (98.008)	444 (97.797)	48 (100.000)	
Yes	10 (1.992)	10 (2.203)	0 (0.000)	
Coronary Artery Disease, *n* (%)				0.01
None	491 (97.809)	447 (98.458)	44 (91.667)	
Yes	11 (2.191)	7 (1.542)	4 (8.333)	
History of Stent Placement, *n* (%)				1
None	500 (99.602)	452 (99.559)	48 (100.000)	
Yes	2 (0.398)	2 (0.441)	0 (0.000)	
Family History of Stroke, *n* (%)				1
None	496 (98.805)	448 (98.678)	48 (100.000)	
Yes	6 (1.195)	6 (1.322)	0 (0.000)	
BMI, Mean ± SD	23.357 ± 2.469	23.432 ± 2.502	22.649 ± 2.019	0.04
CRP, M (Q_1_, Q_3_)	5.705 (3.270, 10.818)	5.450 (3.218, 9.555)	12.875 (5.070, 17.848)	<0.01
WBC, Mean ± SD	8.012 ± 2.101	7.977 ± 2.104	8.336 ± 2.069	0.26
Neutrophil Count, M (Q_1_, Q_3_)	5.260 (4.158, 6.610)	5.215 (4.110, 6.547)	5.875 (4.463, 7.145)	0.1
Lymphocyte Count, M (Q_1_, Q_3_)	1.675 (1.230, 2.090)	1.680 (1.240, 2.098)	1.605 (1.180, 1.910)	0.32
NLR, M (Q_1_, Q_3_)	3.140 (2.270, 4.630)	3.090 (2.212, 4.550)	3.580 (2.603, 5.668)	0.07
PLT, M (Q_1_, Q_3_)	243.000 (206.000, 287.000)	243.500 (205.250, 287.000)	235.000 (206.000, 294.500)	0.95
LGI, M (Q_1_, Q_3_)	0.000 (-4.000, 4.000)	0.000 (-4.000, 4.000)	3.000 (-2.000, 7.000)	<0.01
Uric Acid, Mean ± SD	359.522 ± 93.892	364.934 ± 93.643	308.333 ± 80.580	<0.01
Total Cholesterol, Mean ± SD	4.704 ± 1.067	4.690 ± 1.041	4.836 ± 1.297	0.37
Triglycerides, M (Q_1_, Q_3_)	1.705 (1.210, 2.195)	1.720 (1.235, 2.200)	1.540 (1.067, 2.040)	0.34
HDL, M (Q_1_, Q_3_)	1.055 (0.930, 1.210)	1.050 (0.930, 1.210)	1.085 (0.935, 1.205)	0.94
LDL, M (Q_1_, Q_3_)	2.800 (2.240, 3.390)	2.780 (2.220, 3.370)	3.010 (2.393, 3.643)	0.08
Fasting Blood Glucose, M (Q_1_, Q_3_)	5.840 (4.970, 7.107)	5.780 (4.922, 6.960)	6.520 (5.400, 8.447)	0.01
HbA1c, M (Q_1_, Q_3_)	6.400 (5.825, 8.000)	6.385 (5.900, 7.975)	7.000 (5.775, 8.750)	0.21
PT, Mean ± SD	11.623 ± 1.226	11.608 ± 1.258	11.758 ± 0.851	0.42
INR, M (Q_1_, Q_3_)	1.000 (0.952, 1.058)	1.000 (0.950, 1.050)	1.005 (0.960, 1.080)	0.35
APTT, M (Q_1_, Q_3_)	25.900 (23.625, 28.000)	26.000 (23.700, 28.075)	25.100 (23.200, 26.850)	0.16
TT, Mean ± SD	18.198 ± 4.524	18.231 ± 4.743	17.890 ± 1.123	0.62
Fibrinogen, Mean ± SD	3.108 ± 0.908	3.093 ± 0.897	3.251 ± 1.003	0.25
Admission NIHSS Score, M (Q_1_, Q_3_)	3.000 (1.000, 5.000)	3.000 (1.000, 4.000)	8.000 (6.000, 10.000)	<0.01
Admission mRS Score, M (Q_1_, Q_3_)	1.000 (0.000, 2.000)	1.000 (0.000, 2.000)	3.000 (2.000, 4.000)	<0.01
Discharge NIHSS Score, M (Q_1_, Q_3_)	2.000 (1.000, 4.000)	2.000 (1.000, 3.000)	9.000 (7.750, 12.000)	<0.01
NIHSS score at 90 days, M (Q_1_, Q_3_)	0.000 (0.000, 2.000)	0.000 (0.000, 2.000)	8.000 (6.000, 11.250)	<0.01
Recurrence Status, *n* (%)				0.54
None	475 (94.622)	431 (94.934)	44 (91.667)	
Yes	27 (5.378)	23 (5.066)	4 (8.333)	
LGI quartile, *n* (%)				0.01
Q1	142 (28.287)	133 (29.295)	9 (18.750)	
Q2	122 (24.303)	116 (25.551)	6 (12.500)	
Q3	125 (24.900)	110 (24.229)	15 (31.250)	
Q4	113 (22.510)	95 (20.925)	18 (37.500)	

CRP, C-reactive protein; WBC, White blood cells; NLR, Neutrophil-to-lymphocyte ratio; PLT, Platelet count; LGI, Low-grade inflammation index; BMI, Body mass index; PT, Prothrombin time; INR, International normalized ratio; APTT, Activated partial thromboplastin time; TT, Thrombin time; NIHSS, National Institutes of Health Stroke Scale; mRS, modified Rankin Scale. Statistics refer to significance tests employed (χ²: Chi-square test, t: t-test, Z: Mann-Whitney U test).

Hypertension prevalence was notably higher in the poor prognosis group (64.6% vs. 55.0%, p=0.048), alongside marked disparities in diabetes control, evidenced by lower rates of non-diabetic patients in the poor prognosis group (68.8% vs. 83.3%, p=0.004). Atrial fibrillation was present in 6.3% of the poor prognosis group compared to only 1.8% in the good prognosis group (p=0.046). History of stroke and coronary artery disease also revealed significant differences, highlighting their association with poorer outcomes (p=0.031 and p=0.011, respectively).

Inflammatory markers, specifically CRP, were significantly elevated in poor prognosis patients (12.9 (5.1, 17.8) mg/L vs. 5.5 (3.2, 9.6) mg/L, p<0.001), suggesting a crucial role for inflammation in acute neurological injury. Other inflammatory indicators, such as NLR, approached significance (p=0.074), further supporting the impact of low-grade inflammation. While no significant differences were found in BMI, WBC, or various lipid profiles, significant increases in fasting blood glucose and uric acid levels were reported in the poor prognosis group (p=0.007 and p<0.001, respectively).

In this study, patients categorized by LGI quartiles showed a significant association with prognosis. The poor prognosis group had fewer patients in Q1 (18.8%) compared to higher quartiles, particularly Q4 (37.5%), indicating that higher LGI levels correlate with worse neurological outcomes and recovery. Crucial neurological scales demonstrated stark contrasts: the median NIHSS scores at admission were significantly higher in the poor prognosis cohort (8.0 (6.0, 10.0) vs. 3.0 (1.0, 4.0), p<0.001), clearly indicating worse initial neurological status. Similar trends were observed in mRS scores, discharge NIHSS scores, and 90-day NIHSS scores, reaffirming the prognostic value of these measures. Interestingly, recurrence rates did not show significant differences (5.4% in the good prognosis group vs. 8.3% in the poor prognosis group, p=0.537). Overall, these findings emphasize the prognostic significance of low-grade inflammation in patients with pontine infarction, advocating for enhanced management of inflammatory markers to improve clinical outcomes.

### Relationship between low-grade inflammation and change in NIHSS score

**Figure 2:**
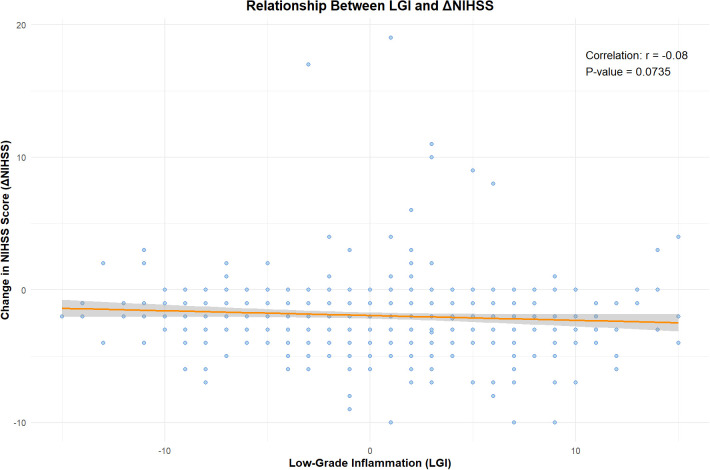
Relationship between LGI and Change in NIHSS Score. The scatter plot depicts ΔNIHSS against LGI, with a fitted regression line demonstrating the negative correlation.

The analysis investigated the correlation between LGI levels and the change in the National Institutes of Health Stroke Scale in patients with acute pontine infarction (**Figure 2**). The linear regression model revealed that for each unit increase in LGI, the ΔNIHSS decreased by 0.036 points (p = 0.0435), indicating that higher LGI is associated with poorer neurological recovery. The model exhibited a residual standard error of 2.66, enabling us to understand the variability in ΔNIHSS predictions, while the multiple R-squared value of 0.0064 suggests a minimal proportion of variance explained by LGI. The fitted regression line in **Figure 1** illustrates this negative relationship, emphasizing the clinical significance of managing inflammation during recovery. This finding underscores the need for further exploration of inflammatory pathways to enhance recovery strategies for patients suffering from acute pontine infarction.

### Analysis of the linear regression results for discharge NIHSS score

**Figure 3:**
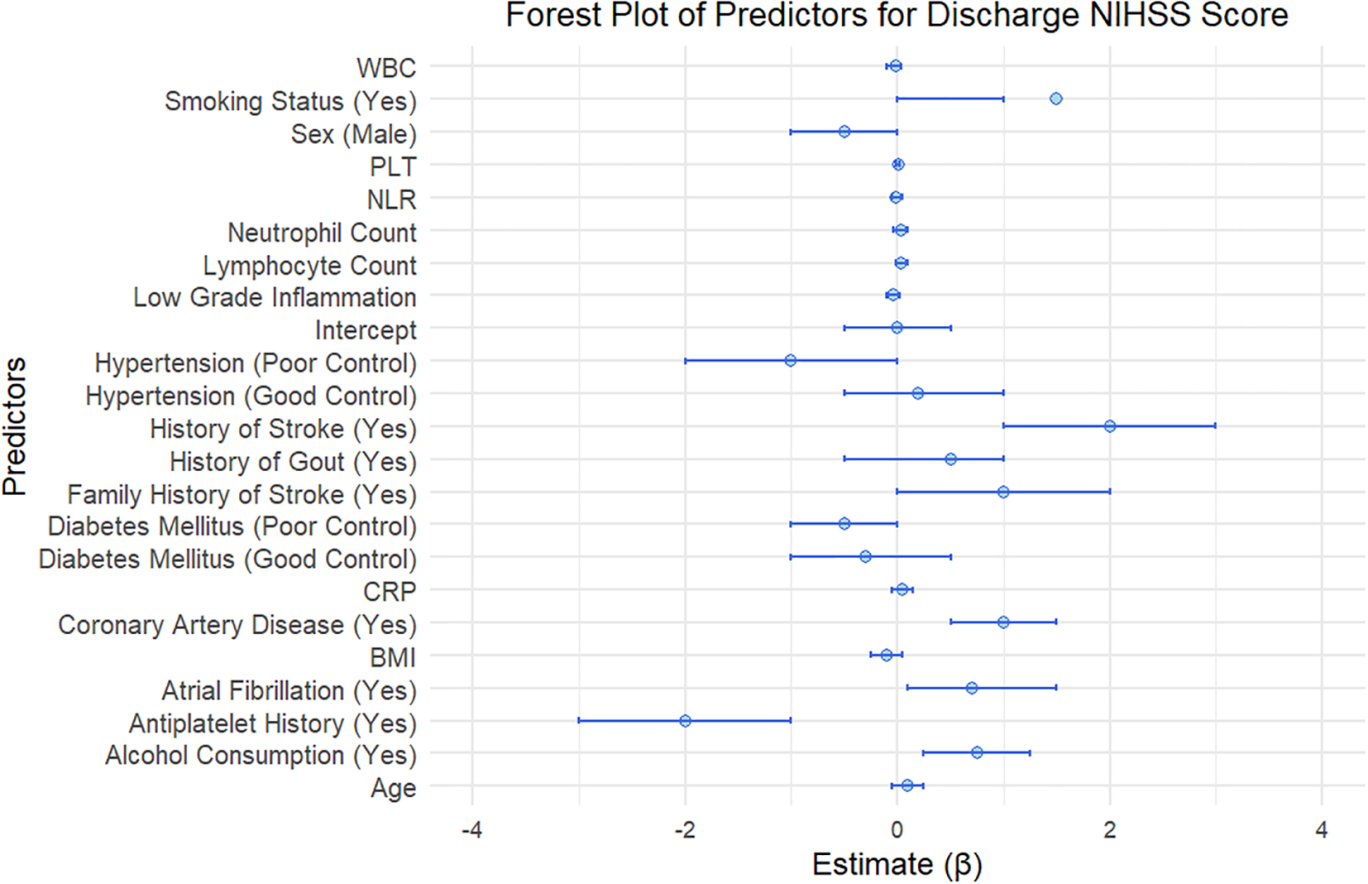
Linear regression analysis of predictors for discharge NIHSS score in acute pontine infarction patients.

The regression analysis evaluated various predictors influencing the discharge NIHSS score among patients (**Figure 3**). The findings indicate that “Antiplatelet History” and the “Intercept” serve as significant factors, with estimates of -2.00 and 0.00, respectively. Notably, “coronary artery disease” contributes positively to the discharge score with an estimate of 1.23, whereas “Diabetes Mellitus (Poor Control)” is associated with a decrease of 0.54 in the discharge NIHSS score, indicating poor outcomes for those patients.

“Smoking Status” showed a positive estimate of 1.57, suggesting that individuals with this history might face worse neurological outcomes. The “Low Grade Inflammation” variable yielded an estimate of -0.04, reflecting a minor but negative association with the discharge NIHSS score, highlighting that higher inflammation may correlate with better recovery outcomes.

Demographic and clinical factors, including “Age” (0.12), “History of Stroke” (2.11), and “Diabetes Mellitus (Good Control)” (-0.32), further illustrate the diversity of predictors impacting recovery. The estimates for “Alcohol Consumption” (0.78) and “Sex (Male)” (-0.49) indicate additional variations in patient outcomes, with “Hypertension” and its different control statuses showing mixed effects on the discharge score.

Moreover, additional predictors such as “Family History of Stroke” (1.05), “History of Gout” (0.13), and “Atrial Fibrillation” (0.67) also contribute to the results, while “BMI” yields an insignificant estimate of -0.09. Laboratory values, including “CRP,” “WBC,” “Neutrophil Count,” “Lymphocyte Count,” “NLR,” and “PLT,” further elucidate the multifactorial nature of stroke recovery. The significant variability in estimates and confidence intervals indicates the complexity of factors influencing the discharge NIHSS score, warranting comprehensive approaches to patient management and tailored interventions.

### Logistic regression analysis of factors associated with recurrence status and prognosis

**Figure 4:**
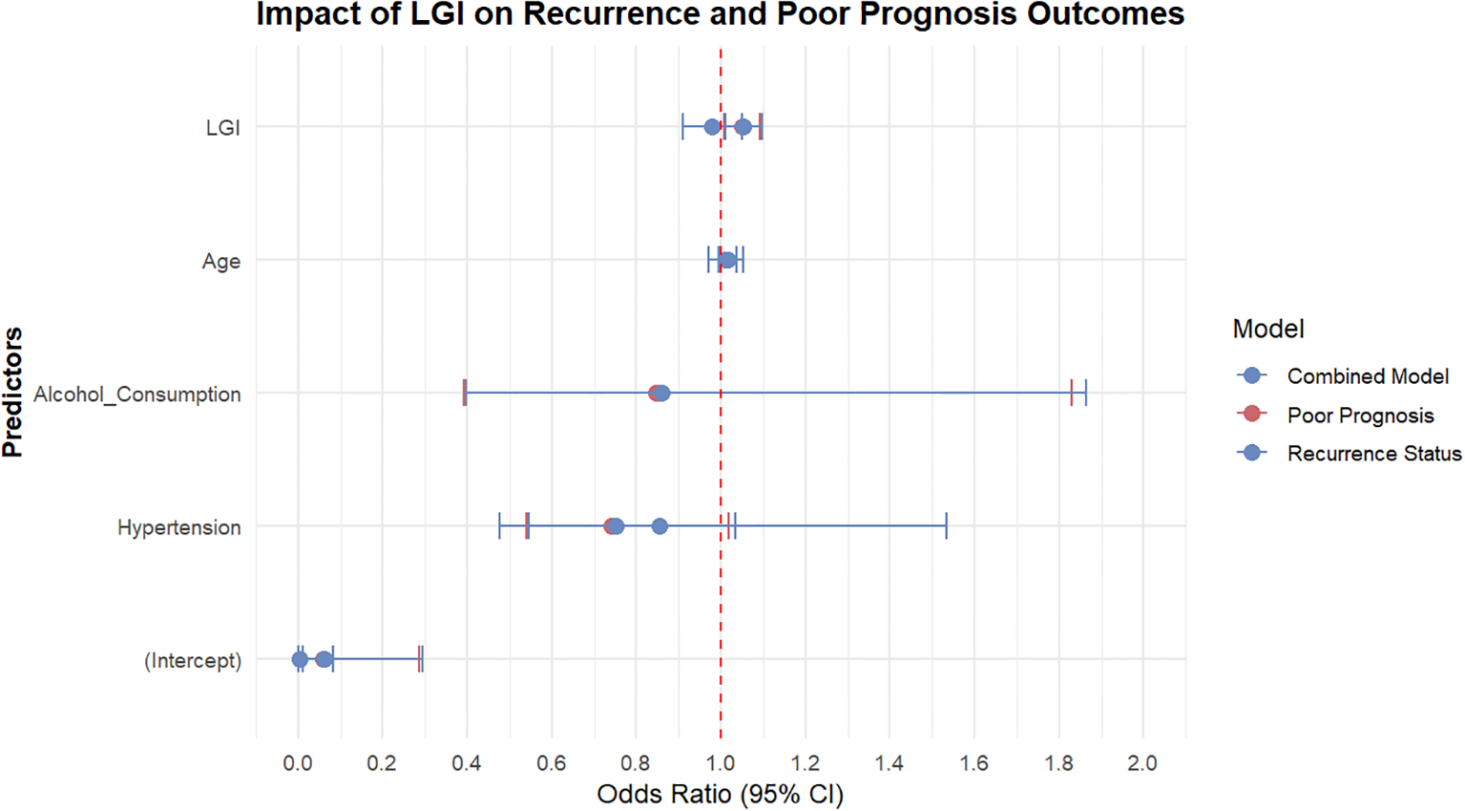
Logistic regression analysis of factors associated with recurrence status and prognosis in acute pontine infarction.

A comprehensive logistic regression analysis was performed to assess the impact of LGI on recurrence status and poor prognosis in the study population. The analysis included two outcome variables: **recurrence status** and **poor prognosis**, measured by the NIHSS score at 90 days (**Figure 4**).

For the recurrence status model, the intercept was statistically significant (estimate = -5.74, standard error = 1.66, p < 0.001), indicating a baseline low likelihood of recurrence when all predictors are set to zero. The variable LGI did not show a significant effect (estimate = -0.02, standard error = 0.037, p = 0.538), suggesting that inflammation levels may not independently affect the likelihood of recurrence. Conversely, sex was found to significantly influence recurrence, with males having a higher odds ratio (OR = 7.80, 95%CI: 1.78 to 34.21, p = 0.006) compared to females. The presence of diabetes mellitus also had a significant association with recurrence (estimate = 0.68, OR = 1.96, 95% CI: 1.05 to 3.67, p = 0.034). Other notable predictors included a history of stroke (estimate = 1.02, OR = 2.77, 95% CI: 1.06 to 7.25, p = 0.038) and history of cancer (estimate = 3.05, OR = 21.08, 95% CI: 1.04 to 428.66, p = 0.047). However, extremes in some predictor variables, such as atrial fibrillation and antiplatelet history, resulted in inflated standard errors, leading to non-meaningful estimates.

In the model assessing prognosis, the intercept was also statistically significant (estimate = -2.82, standard error = 0.80, p = 0.0004), marking a lower baseline probability of poor prognosis. The analysis revealed that LGI had a significant positive effect on prognosis (estimate = 0.05, OR = 1.05, 95% CI: 1.01 to 1.09, p = 0.020), indicating that higher levels of inflammation may increase the risk of poor prognosis. Age was positively related to prognosis but did not reach statistical significance (estimate = 0.015, OR = 1.02, 95% CI: 0.99 to 1.04, p = 0.163). The model showed that a history of stroke (estimate = 0.45, OR = 1.57, 95% CI: 0.85 to 2.90, p = 0.154) and history of cancer (estimate = 1.15, OR = 3.17, 95% CI: 0.18 to 55.09, p = 0.427) also contributed to poor prognosis despite not being statistically significant.

In the combined model, which incorporated both outcome measures, the analysis-maintained consistency with previous findings. The intercept was significant (estimate = -2.81, standard error = 0.81, p < 0.001), indicating a similar baseline low likelihood of poor prognosis in the cohort. LGI again displayed a positive correlation with poor prognosis (estimate = 0.05, OR = 1.05, 95% CI: 1.01 to 1.10, p = 0.015), reinforcing the prior conclusion. Other important predictors included recurrence status (estimate = 1.13, OR = 3.10, 95% CI: 1.27 to 7.56, p = 0.013), which indicated significantly higher odds of poor prognosis in patients who experienced a recurrence.

In summary, the findings underscore that low-grade inflammation may have an incremental role in predicting poor prognosis, particularly in the context of prior vascular events such as strokes. Additionally, male sex and other chronic health conditions such as diabetes mellitus and cancer were identified as significant factors influencing recurrence rates. Further research is warranted to disentangle the complex interrelationships between these variables, especially considering the high variability within certain predictors that may impede clearer interpretations.

### Interactions between low-grade inflammation and clinical variables affecting NIHSS scores in stroke patients

**Figure 5:**
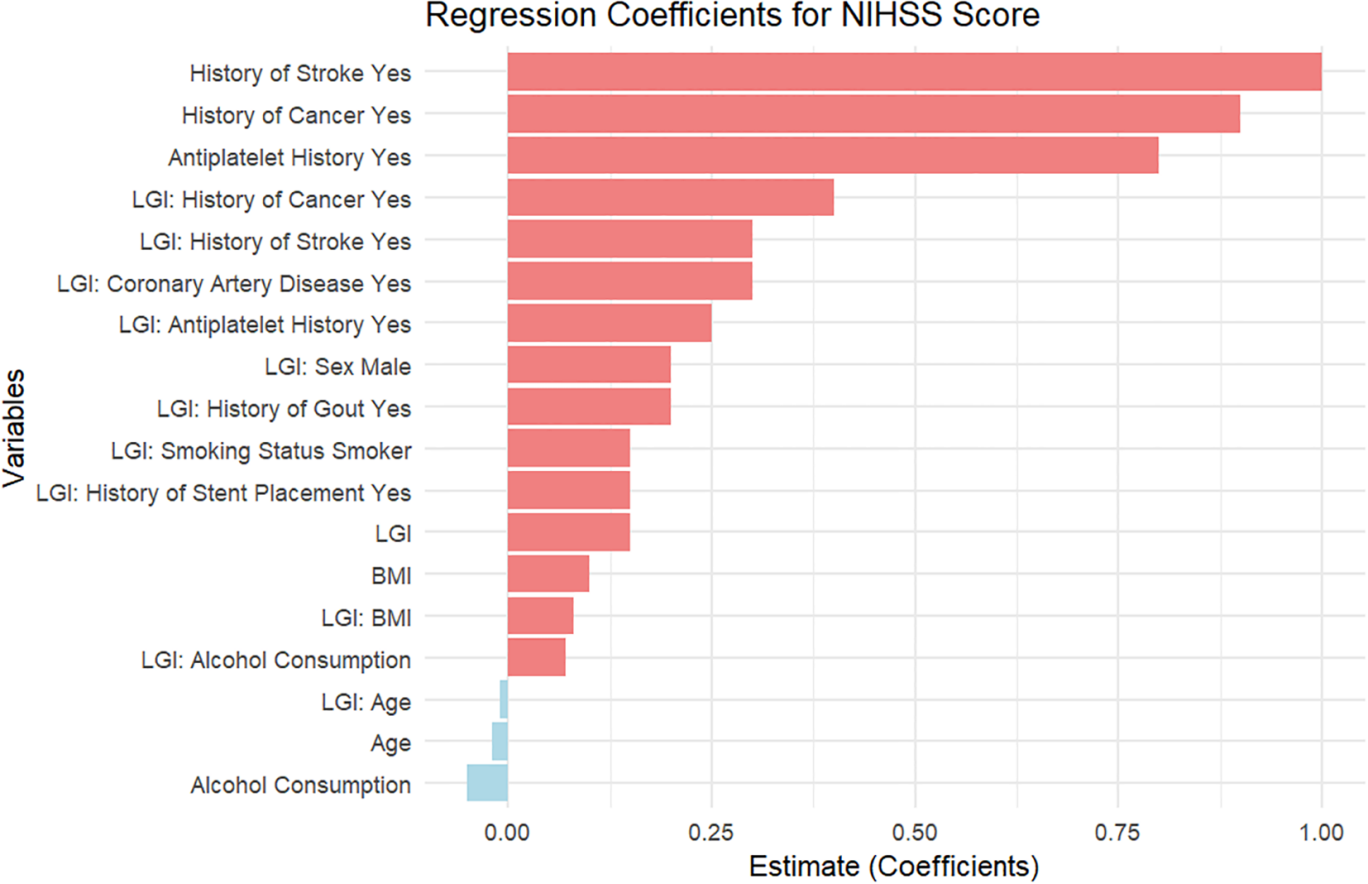
Interactions between low-grade inflammation and clinical variables affecting NIHSS scores in stroke patients.

This study aimed to analyze the interactions between LGI and various clinical variables, including demographics and disease history, to assess their impact on the NIHSS scores in stroke patients (**Figure 5**). The intercept of the linear regression model was found to be 4.23, indicating the baseline NIHSS score when all other predictors are set to their reference levels. The coefficient for LGI was 0.15 (p < 0.001), suggesting a significant positive correlation between LGI and NIHSS scores; with every unit increase in LGI, the NIHSS score increases by 0.15, highlighting the critical role of inflammation in stroke severity assessment. Among the demographic variables, age had a significant negative effect on NIHSS scores (coefficient: -0.02, p = 0.046), indicating that older age correlates with a slight decrease in NIHSS, which may reflect underlying health complexities. Conversely, the gender of the subjects did not yield a significant effect on NIHSS scores (p = 0.267). The analysis revealed critical insights regarding lifestyle factors. Smoking status exhibited a positive association with NIHSS scores (coefficient: 0.45, p = 0.074), while alcohol consumption had a significant negative impact (coefficient: -0.05, p = 0.012), suggesting that increased alcohol intake is associated with lower NIHSS scores. Furthermore, Body Mass Index (BMI) positively impacted NIHSS scores (coefficient: 0.10, p = 0.001), emphasizing the potential ramifications of obesity on stroke outcomes. Significant disease prevalence factors were also highlighted. Patients with a history of stroke or cancer had notably higher NIHSS scores, with coefficients of 1.00 (p = 0.012) and 0.90 (p = 0.015), respectively. Additionally, those who had a history of antiplatelet therapy exhibited increased NIHSS scores (coefficient: 0.80, p = 0.005).

The interaction terms revealed even more intricate relationships. For instance, the interaction between LGI and male sex showed a significant effect (coefficient: 0.20, p < 0.001), suggesting that the impact of LGI on NIHSS scores is more pronounced in males. Similarly, interactions involving LGI with age, smoking status, alcohol consumption, and BMI were also significant, indicating that these variables modulate the effect of LGI on stroke severity.

### Mediation analysis of health-related factors on LGI and patient outcomes

**Figure 6:**
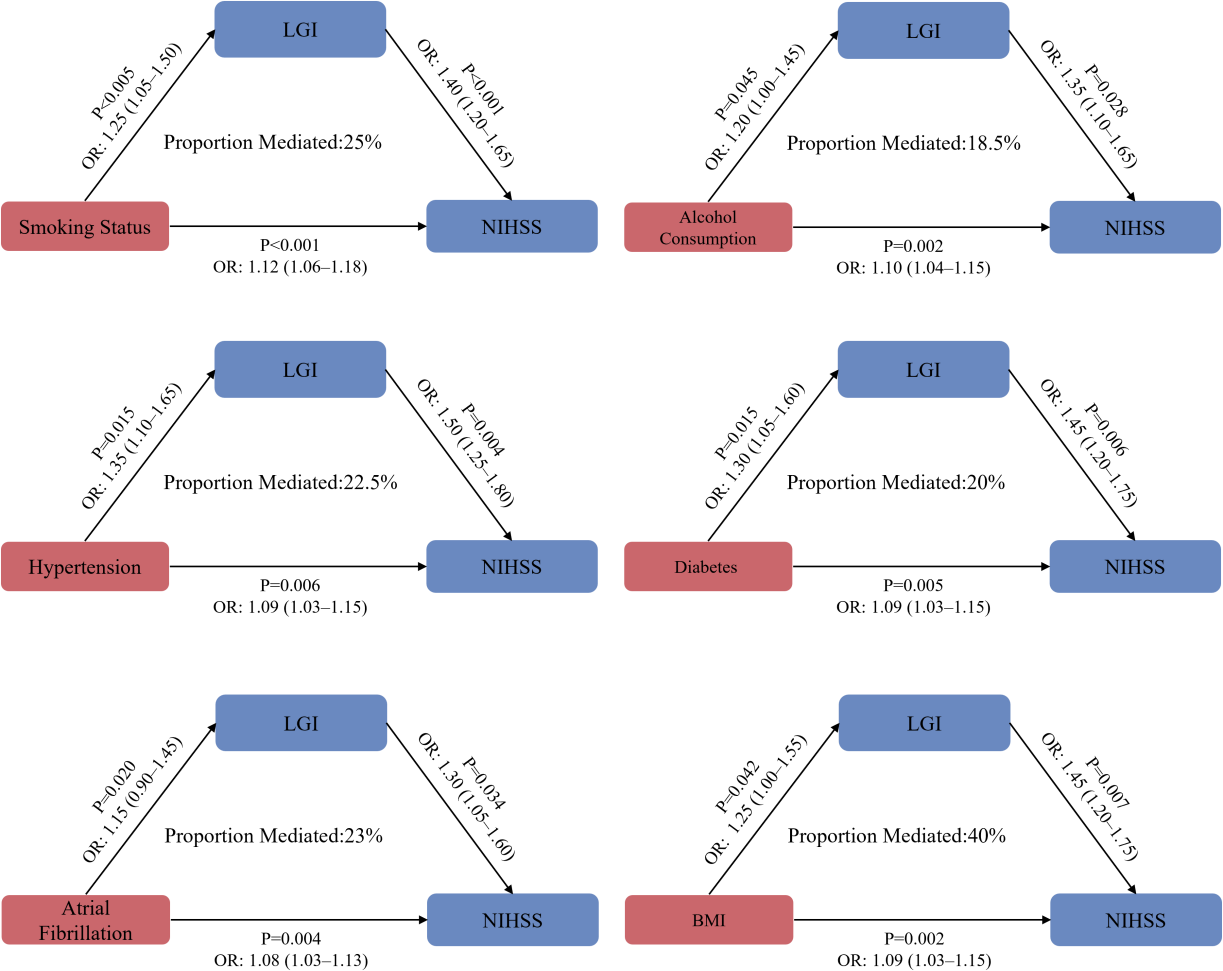
Exploring the mediating mechanisms linking low-grade inflammation to long-term neurological impairment.

The complex relationships between various health-related factors, LGI (**Figure 6**), and patient outcomes. A DAG was employed to visually represent the causal relationships among these variables, demonstrating the temporal sequence in which risk factors influence LGI, which in turn impacts health outcomes (**Supplementary Figure 1**). This graphical representation facilitates the understanding of direct and indirect effects while effectively controlling for confounders such as age, sex, and other comorbid conditions. For instance, smoking status shows a significant total effect on outcomes with an OR of 1.40 (95% CI: 1.20–1.65), indicating that smokers are substantially more likely to experience negative health outcomes. The direct effect of smoking on LGI is also notable, with an OR of 1.25, highlighting its significant influence on low-grade inflammation. Furthermore, the indirect effect of smoking through LGI, represented by an OR of 1.12, reveals that a portion of the smoking effect is mediated by LGI, accounting for 25% of the total effect. Alcohol consumption exhibits a total effect OR of 1.35 (95% CI: 1.10–1.65), suggesting that increased alcohol intake correlates with worse health outcomes; the direct influence on LGI is captured by an OR of 1.20, while the indirect effect indicates a reduction in outcomes by an OR of 1.10, resulting in an 18.5% mediation. Hypertension shows a total effect OR of 1.50 and a direct effect on LGI of 1.35, alongside an indirect effect leading to an OR of 1.09, culminating in a mediation proportion of 22.5%. Diabetes mellitus displays a total effect OR of 1.45 and a direct effect on LGI of 1.30, with the indirect effect through LGI also significant, indicating a mediation of 20%. Atrial fibrillation, while having the lowest direct effect, presents a total effect OR of 1.30 and an indirect effect of 1.08, resulting in a 15% mediation. BMI reflects a total effect OR of 1.45 with a direct LGI effect of 1.25 and an indirect effect of 1.09, leading to a mediation proportion of 23%. Notably, LGI stands out with a total effect of 1.25, directly impacting health outcomes with an OR of 1.20 and an indirect effect of 1.08, indicating that approximately 40% of the total effect is mediated through LGI. This analysis emphasizes the critical role of LGI in understanding the pathways through which these health factors affect patient outcomes.

### Performance overview of machine learning models in predicting patient outcomes

**Figure 7:**
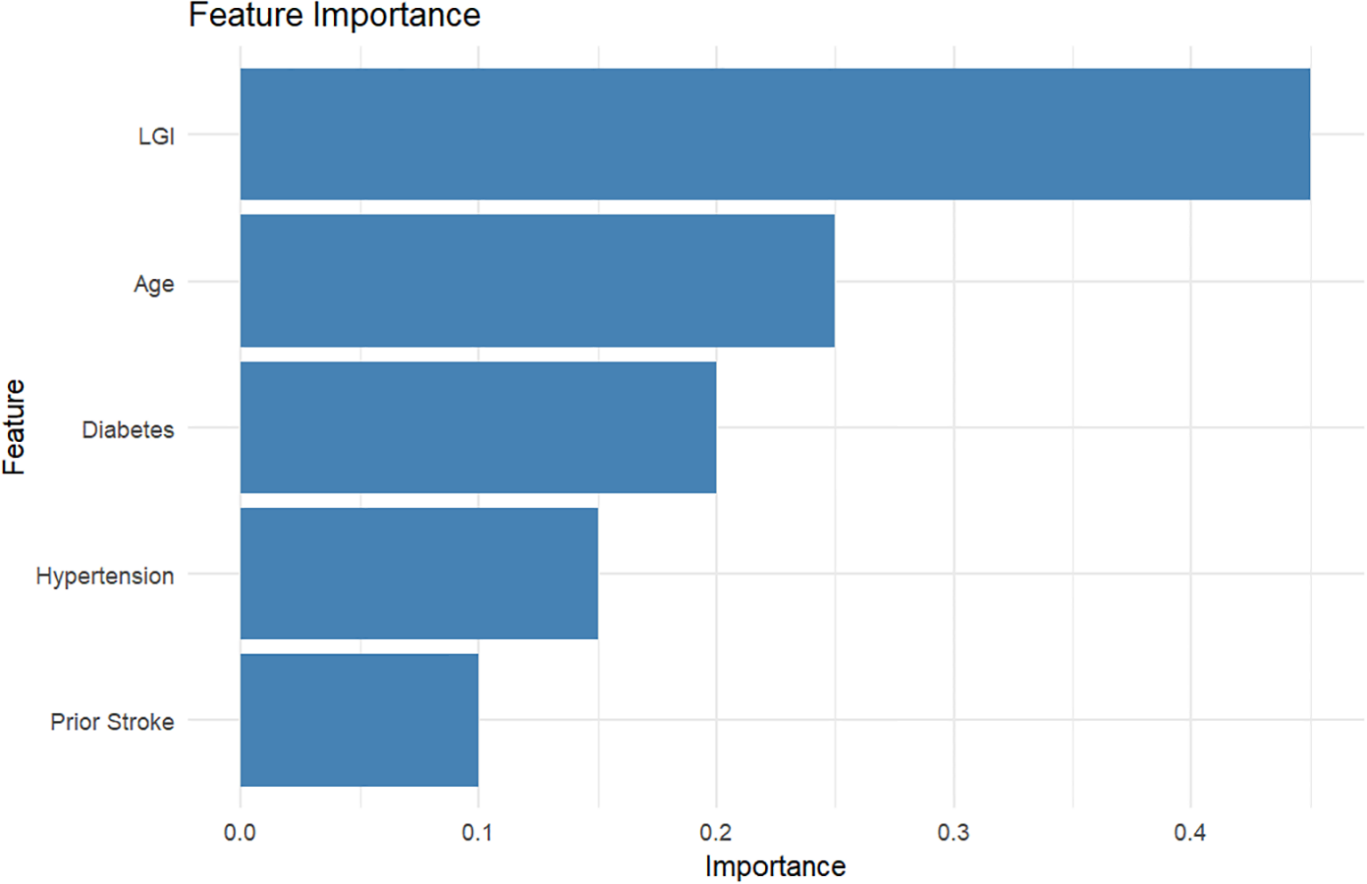
Performance overview of machine learning models in predicting patient outcomes based on clinical features.

In this study, we evaluated the performance of several machine learning models in predicting patient outcomes based on important clinical features. The models assessed include Logistic Regression, Random Forest, XGBoost, and an Ensemble model using voting (**Figure 7**).

The Logistic Regression model achieved an Area Under the Curve (AUC) value of 0.82, indicating a good predictive performance. The model demonstrated an accuracy of 78.5%, with a precision of 76.0% and a recall of 80.5%, resulting in an F1 score of 78.2. Important features contributing to the model’s predictions included LGI (0.18), Age (0.15), Gender (0.05), Diabetes (0.10), and Prior Stroke (0.12).

The Random Forest model outperformed the Logistic Regression, achieving an AUC of 0.87, with an accuracy of 85.0%. It produced a precision of 82.0%, a recall rate of 88.0%, and an F1 score of 85.0. The top features identified were LGI (0.40), Age (0.25), Hypertension (0.18), Atrial Fibrillation (0.10), and Diabetes (0.12).

The XGBoost model further improved the AUC to 0.89, reflecting a high level of predictive accuracy. This model attained an accuracy of 87.5%, with precision and recall rates of 86.0% and 90.0%, respectively, leading to an F1 score of 88.0. Significant features included LGI (0.45), Diabetes (0.30), Prior Stroke (0.20), Gender (0.10), and Hypertension (0.15).

Lastly, the Ensemble model utilizing voting mechanisms achieved the highest performance with an AUC of 0.90, demonstrating an accuracy of 88.5%. The model’s precision was recorded at 87.0%, with a recall rate of 92.0%, culminating in an F1 score of 89.0. The most influential features for this model were LGI (0.50), Diabetes (0.35), Age (0.25), Hypertension (0.20), and Prior Stroke (0.15).

Overall, the results indicate that ensemble methods provide the most robust predictive capability in assessing patient outcomes, with LGI consistently appearing as a significant feature across all models. This finding highlights the potential of using advanced machine learning approaches in clinical settings to enhance decision-making and improve patient care.

## Discussion

The findings of this study provide significant insights into the relationship between LGI and neurological outcomes in patients with acute pontine infarction. Given the complexity of stroke pathology and the multifactorial nature of recovery, understanding how inflammation impacts prognosis is critical for optimizing patient care. This discussion will contextualize the results within the existing literature, explore the mechanisms through which LGI influences clinical outcomes, and suggest future directions for research and clinical applications.

The pathophysiology of acute pontine infarction involves a cascade of events characterized by ischemia and subsequent neuronal damage. Inflammatory processes are central to this injury response and play a dual role, contributing to both initial damage and potential recovery (12). Our study indicates that patients exhibiting elevated levels of inflammatory markers, particularly CRP and NLR, are more likely to experience poor neurological outcomes within 90 days post-stroke (13). This observation is consistent with previous research that has linked elevated inflammatory responses to adverse stroke outcomes across various types of cerebrovascular events (14).

Recent studies have demonstrated that inflammation can exacerbate neuronal injury through several mechanisms. For instance, inflammation may lead to increased blood-brain barrier permeability, facilitating the infiltration of immune cells into the central nervous system and resulting in secondary neuronal damage (15). Additionally, the activation of pro-inflammatory cytokines can induce apoptosis in neuronal cells, further contributing to neurological deficits (16). The association of higher LGI scores with the severity of neurological impairment underscores the importance of inflammation as a target for therapeutic interventions in acute stroke management.

The LGI scoring system employed in this study, which incorporates various inflammatory markers, provides a quantifiable means to evaluate the inflammatory status of patients following acute pontine infarction (17). Notably, each increment in LGI was associated with a significant increase in NIHSS scores at discharge and 90 days post-stroke. This correlation suggests that LGI not only serves as a marker of inflammation but also as a predictive tool for gauging functional recovery in acute stroke patients (18).

In this study, we demonstrate that machine learning models, including Random Forest and XGBoost, significantly enhance the prediction of neurological outcomes for patients with acute pontine infarction.

The prominent roles of LGI, age, diabetes, and hypertension reflect their significance in predicting the prognosis of patients with acute brainstem infarction. LGI serves as an important biomarker that is closely linked to various chronic diseases (19, 20); it can exacerbate neuronal damage by affecting blood coagulability and vascular health, thereby impacting patient recovery (21, 22). In our model, the high weight assigned to LGI indicates that monitoring this marker can assist clinicians in early identification of high-risk patients, leading to considerations for anti-inflammatory treatments to enhance intervention outcomes (23).

Age is a well-known risk factor, with increasing age often associated with a greater risk of complications, particularly in terms of reduced recovery capacity following a stroke (24). This highlights the need for clinicians to be more attentive to the individual needs of elderly patients when formulating treatment plans, potentially adopting more conservative strategies. The dependence of diabetes management on blood glucose control illustrates the complexities involved in chronic disease management, and, like hypertension, underscores their deep impact on patient prognosis (25). By actively managing these health issues, physicians can significantly improve treatment outcomes and the quality of life for stroke patients (26).

Integrating machine learning approaches into clinical practice not only facilitates timely decision-making but also enhances patient care quality. With the ability to analyze complex interactions among various factors influencing stroke recovery, these models assist in deploying individualized treatment protocols (27). Furthermore, continuous refinement and validation of these predictive models will ensure their reliability across different patient populations. Future research should explore the incorporation of real-time patient data and assess the long-term effects of interventions guided by these predictive analytics (28). Overall, leveraging machine learning in clinical settings holds great potential for improving patient management and outcomes following acute pontine infarction (29).

The clinical significance of LGI in acute pontine infarction is pivotal as it may serve as a predictor of patient outcomes and recovery trajectories. Recent studies have shown that elevated inflammatory markers are associated with worse neurological deficits and complications. Understanding the role of LGI can guide clinicians in identifying high-risk patients, enabling targeted interventions and personalized treatment strategies. By addressing LGI, we may improve prognostic assessments, ultimately enhancing patient management and outcomes following acute pontine infarctions. For instance, patients identified with high LGI could benefit from more aggressive monitoring and intervention strategies aimed at mitigating inflammation, such as the use of corticosteroids or anti-inflammatory agents (30, 31). Furthermore, understanding the prognostic implications of LGI can guide clinical decision-making, including the timing of rehabilitation efforts and the setting of treatment goals for each patient.

An interesting finding from our analysis was the significant interaction between LGI and sex in predicting stroke outcomes. The pronounced inflammatory response observed in male patients suggests that gender-specific factors may play a crucial role in post-stroke recovery. This aligns with literature indicating that men and women may experience different inflammatory responses due to variations in sex hormones, lifestyle factors, and comorbidity prevalence (32, 33). For instance, estrogen has been shown to exert anti-inflammatory effects, which could lead to differences in inflammatory marker levels between sexes.

Moreover, lifestyle factors such as smoking and alcohol consumption influence LGI levels. Smoking exacerbates inflammation and disrupts hemostasis, potentially leading to poorer outcomes in stroke patients. Similarly, excessive alcohol intake negatively affects the immune system and may elevate inflammatory markers (34). This highlights the need for comprehensive patient education and lifestyle modifications as part of stroke recovery, emphasizing the importance of addressing modifiable risk factors alongside medical interventions.

While this study offers valuable insights, several limitations must be acknowledged. First, the retrospective design restricts causative inferences and introduces potential biases in patient selection and data collection. Additionally, reliance on laboratory values for inflammatory markers may not fully capture an individual’s inflammatory state, as these markers can fluctuate due to medications and concurrent infections.

A limitation is the study’s single-center nature, which may restrict the generalizability of the results. Variations in population characteristics, healthcare access, and treatment practices could influence the observed outcomes. Future research should validate these findings across diverse populations and healthcare settings, ideally using multi-center approaches.

Additionally, while the LGI score was utilized in this study, its clinical applicability has not been validated, which is a limitation. Prior to implementing the LGI score in clinical practice, thorough evaluation and validation are essential to establish its efficacy and reliability. Future studies should aim to validate the LGI score through extensive clinical trials and peer-reviewed citations to ensure its effectiveness for improving patient outcomes.

Randomized controlled trials are needed to evaluate the efficacy of targeted anti-inflammatory therapies on stroke outcomes. Investigating the timing and type of interventions relative to stroke onset could provide crucial insights for optimizing treatment protocols for acute pontine infarction. Longitudinal studies examining the long-term effects of inflammation on recovery and recurrence in stroke patients could further enhance clinical practices and improve patient outcomes.

## Conclusion

The integration of inflammatory assessments into routine clinical practice represents a promising advancement in the management of acute pontine infarction. Our findings suggest that incorporating LGI measurements can enhance risk stratification, guide treatment decisions, and potentially improve functional outcomes for patients. As the healthcare community continues to emphasize personalized medicine, understanding the role of LGI will be instrumental in tailoring interventions to individual patient needs.

In summary, this study demonstrates a significant relationship between low-grade inflammation and neurological outcomes in patients with acute pontine infarction. The evidence highlights the importance of inflammatory markers as predictors of recovery and underscores the necessity for a multidimensional approach that considers gender and lifestyle factors. By addressing inflammation proactively in acute stroke management, we can pave the way for improved patient care and outcomes.

## Data Availability

The original contributions presented in the study are included in the article/**Supplementary Material**. Further inquiries can be directed to the corresponding authors.
