# Supplementary figures and images for: Impact of low-grade inflammation on neurological outcomes in patients with acute pontine infarction: a retrospective cohort study
Source: Front Immunol. 2026 Jan 13;16:1744943. doi: 10.3389/fimmu.2025.1744943 (PMC12835896; doi:10.3389/fimmu.2025.1744943)

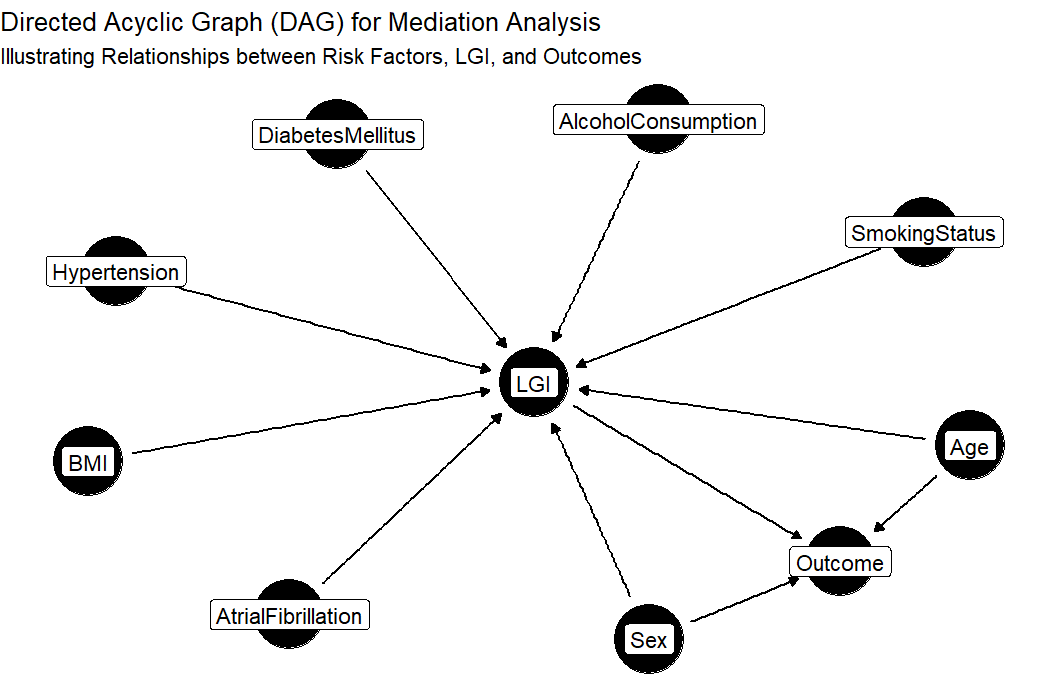

Supplement: Supplementary file 1 [file Image1.tiff]
